# Mitigating In-Column Artificial Modifications in High-Temperature LC–MS for Bottom–Up Proteomics and Quality Control of Protein Biopharmaceuticals

**DOI:** 10.1021/acs.analchem.4c02819

**Published:** 2024-08-28

**Authors:** Mykyta R. Starovoit, Siddharth Jadeja, Taťána Gazárková, Juraj Lenčo

**Affiliations:** Department of Analytical Chemistry, Faculty of Pharmacy in Hradec Králové, Charles University, Heyrovského 1203/8, 500 03 Hradec Králové, Czech Republic

## Abstract

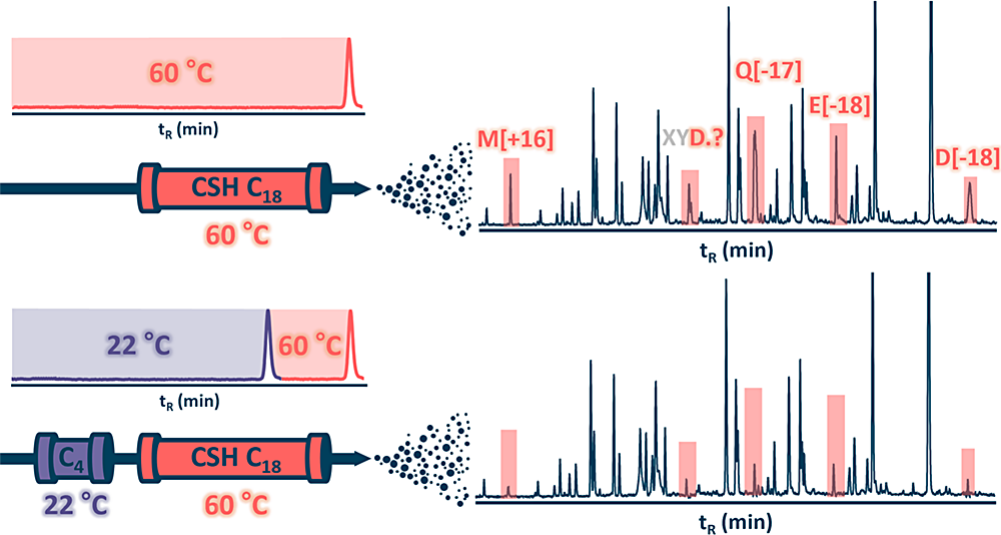

Elevating the column temperature is an effective strategy for improving the chromatographic separation of peptides. However, high temperatures induce artificial modifications that compromise the quality of the peptide analysis. Here, we present a novel high-temperature LC–MS method that retains the benefits of a high column temperature while significantly reducing peptide modification and degradation during reversed-phase liquid chromatography. Our approach leverages a short inline trap column maintained at a near-ambient temperature installed upstream of a separation column. The retentivity and dimensions of the trap column were optimized to shorten the residence time of peptides in the heated separation column without compromising the separation performance. This easy-to-implement approach increased peak capacity by 1.4-fold within a 110 min peptide mapping of trastuzumab and provided 10% more peptide identifications in exploratory LC–MS proteomic analyses compared with analyses conducted at 30 °C while maintaining the extent of modifications close to the background level. In the peptide mapping of biopharmaceuticals, where in-column modifications can falsely elevate the levels of some critical quality attributes, the method reduced temperature-related artifacts by 66% for N-terminal pyroGlu and 63% for oxidized Met compared to direct injection at 60 °C, thus improving reliability in quality control of protein drugs. Our findings represent a promising advancement in LC–MS methodology, providing researchers and industry professionals with a valuable tool for improving the chromatographic separation of peptides while significantly reducing the unwanted modifications.

LC–MS allows for a comprehensive analysis of proteins following their cleavage to peptides using a sequence-specific protease. This so-called bottom-up approach has become a mainstream approach in proteomics.^1^ It is also increasingly accepted for multiattribute analyses of protein biopharmaceuticals, where this approach is referred to as peptide mapping.^2^

Because of their physicochemical properties, separation of peptides using reversed-phase liquid chromatography (RPLC) within LC–MS analyses requires specific considerations.^3^ Elevation of the column temperature represents one of the most efficient and accessible means for improving the quality of peptide separation. High temperature enhances peptide diffusivity and their adsorption–desorption kinetics on the RPLC stationary phase, which results in narrower peaks with increased intensity.^4^ Certain peptide subsets may particularly profit from the high column temperature, such as hydrophobic peptides due to improved recovery^5,6^ and peptides with vicinal prolines because of enhanced kinetics of *cis–trans* isomerization of the corresponding peptide bond.^4,7^ High temperature reduces the mobile phase viscosity, lowering the back pressure. Finally, the elution strength of the mobile phase is greater at high temperature,^8^ which reduces the consumption of organic solvents.^9^

However, high-temperature liquid chromatography (HTLC) has an important drawback. Because of exposure to a high temperature and an acidic mobile phase, peptides undergo artificial modifications during retention in the separation column. The typical temperature-related modifications include pyroGlu formation from the N-terminal Glu or Gln, Asp dehydration, and hydrolysis of peptide bonds at Asp.^4^ In addition, N-terminal Cys alkylated using iodoacetamide can undergo cyclization.^10^ Certain dependence on column temperature was also observed for Met oxidation,^4,8^ although most studies discuss the effect of residual metals and the column age.^11−13^ The formation of the artifacts reduces the signal of unmodified parent peptides, decreasing the chance for their identification. In data-dependent proteomic analyses, modified peptides providing no additional biological information compete for sequencing with unmodified peptides. In data-independent analyses, peptide artifacts increase the complexity of the product spectra, complicating their evaluation. Furthermore, some analysis-related modifications can mimic in vivo modifications, hampering their analysis.^14,15^ More importantly, in the quality control of protein biopharmaceuticals, artificial modifications can be formed at the sites that are critically important for drug efficacy or safety. They cannot be distinguished from the modifications originating during protein drug production and processing, which represent the real manufacturer’s interest. Thus, temperature-induced artifacts may falsely increase the extent of modification at the sites determined as critical quality attributes (CQA). Consequently, quantifying some specific modifications using HTLC is unreliable. Therefore, these analyses should be carried out at a low column temperature, which is, however, not optimal for separation quality.

Here, we sought to develop a high-flow HTLC–MS method for peptide analysis that would still profit from the high column temperature while mitigating artificial modifications to a maximum extent. Inspired by our recent findings on the dependence of artifact formation on peptide in-column residence time,^4^ we leveraged the protective effect of an inline trap column installed upstream of the separation column. Trap columns are customary in nanoflow LC–MS, which dominates bottom-up proteomics, where they are installed in a way that reduces the time needed to deliver the sample from the autosampler. This is not needed in the higher-flow regimes that are gaining broader interest in proteomics and are standard for peptide mapping of protein biopharmaceuticals due to higher throughput, robustness, and longer column lifetimes.^16−20^ Short guard columns are recommended to be installed on the separation columns used for analytical flow regimes to extend their lifetime.^21^ Regardless of the regime, the trap or guard columns have been installed for reasons other than suppression of unwanted modifications. They are typically maintained at the same temperature as the separation column, yet manipulating the trap column temperature can improve proteome coverage.^22^

In our method, the trap column is maintained at a lower temperature to keep peptides safe for a considerable amount of time before they finally elute from the separation column. Thanks to a less retentive stationary phase of the trap column, peptides elute from it at a concentration of acetonitrile that still allows their refocusing at the head of the separation column maintained at a high temperature for superior separation performance. Consequently, peptides spend only a short time in the heated separation column but still fully exploit its intrinsic separation efficiency. We compared the optimized trap-elute setups to direct injections using a range of peptide samples of various complexities. Our method reduced the extent of artifact formation and increased the number of identified peptides in exploratory LC–MS analyses of complex proteomic samples. Crucially, the trap-elute setup significantly decreased the level of artificial modifications in analyses of four protein biopharmaceuticals, with the most pronounced effect observed for Met oxidation and pyroGlu formation.

## Experimental Section

### Chemicals and Reagents

Unless otherwise stated, chemicals and reagents were purchased from Sigma-Aldrich/Merckin the highest available grade. LC–MS grade solvents and additives for mobile phases were purchased from Merck or Honeywell. Trypsin Platinum Mass Spec grade was purchased from Promega, and 1 M Tris-HCl buffer, pH 7.5, was purchased from SERVA. iRT peptides were synthesized by Royobiotech (China).^23^ Unused leftovers of freshly reconstituted trastuzumab (Herceptin, Roche), bevacizumab (Avastin, Roche), aflibercept (Zaltrap, Sanofi), and durvalumab (Imfinzi, AstraZeneca) were received from Multiscan Pharma, Czech Republic. Sample preparation is described in the Supporting Information (Note S1).

### Trap and Separation Columns

Separation columns Acquity Premier 2.1 × 150 mm and Acquity UPLC 1 × 150 mm packed with 1.7 μm CSH C_18_ 130 Å particles (Waters) were used in the study. Candidate trap columns of 2.1 mm i.d. included 30 and 10 mm Accucore 150-C_4_ columns packed with 2.6 μm superficially porous 150 Å particles (Thermo Fisher Scientific), and 5 mm Acquity UPLC VanGuard precolumns packed with 1.7 μm Protein BEH C_4_ 300 Å particles or 1.7 μm BEH C_8_ 130 Å particles (Waters), respectively. The VanGuard precolumns were installed through a zero-dead-volume union. For the microflow separation, we examined two marketed 1 × 5 mm EXP Trap cartridges packed with 5 μm Opti-Sil C_4_ 300 Å silica particles or polymer-based 10 μm PLRP-S 300 Å particles, respectively (Optimize Technologies). The 1 × 5 mm EXP Trap cartridge was also packed with 1.7 μm Protein BEH C_4_ 300 Å particles by the hardware manufacturer. All columns were conditioned by injections of a complex peptide mixture to achieve reproducible peak shapes and retention times.

### Instruments

An Agilent 1290 Infinity II LC-UV system was used for experiments that did not require the identification of the analytes. The separation column was connected to the autosampler using a 0.1 mm i.d. Viper capillary equipped with a 1 μL passive preheater (Thermo Fisher Scientific) placed in a separation column thermostat. In the trap-elute setup, the trap column was installed downstream of the passive preheater and connected to the separation column with an additional capillary. The maximum portion of this capillary was placed in the column thermostat. The preheater and the trap column were placed in an external thermostat SICO-300 (SISw, Czech Republic).

LC–MS analyses were carried out using a Vanquish Horizon UHPLC system hyphenated to a Q Exactive HF-X mass spectrometer (Thermo Fisher Scientific) in positive ion mode. The separation column was connected with the autosampler via a 0.1 × 550 and 0.1 × 380 mm Viper capillary joined with a Viper union (Scheme S1). The shorter capillary with an active preheater was placed in the separation column thermostat. In the trap-elute setup, the Viper union was replaced with a trap column. The trap column and the maximum length of the 0.1 × 550 mm capillary were placed in the SICO-300 thermostat. Components A and B of the mobile phase were water and acetonitrile, respectively, acidified with 0.05% formic acid.

The flow rate of the mobile phase was 300 μL/min in experiments involving 2.1 mm i.d. columns. The flow rates in microflow experiments were 68 and 50 μL/min for the trap column optimization and analyses of the Jurkat cell digest, respectively. Eluted peptides were electrosprayed using a 0.003 in. spray needle inserted in a HESI-II probe. The ionization in LC–MS analyses of the Jurkat cells digest was enhanced via nebulization of the eluate with nitrogen enriched with vapors of 100% propionic acid, which were obtained using a homemade vaporizer similar to that previously published.^24^ Detailed ion source settings and settings of the mass analyzer for each experiment are specified in the Supporting Information (Tables S1 and S2). All analyses were performed in triplicate. All LC–MS files were deposited in the ProteomeXchange repository with the identifier PXD052716.^25^

### Trap-Elute Setup Optimization

A half microliter of sample containing uracil, acetophenone, toluene, and naphthalene (HPLC Prodigy Standard AL0-3045, Phenomenex) was isocratically separated at 30% component B using direct injection in the 2.1 mm i.d. separation column at 70 °C and the trap columns at 22 °C for the investigation of column retentivity. Analytes were detected at 254 nm.

One microliter of iRT peptide mixture was separated using 10 and 30 min linear gradients of 1–41% component B using direct injection in the separation columns, the trap-elute setups, and the trap columns kept at the temperatures described above. Peptides were detected at 214 nm.

### Artificial Modifications in Model LC–MS Analyses with Various Isocratic Hold Times

Ten micrograms of the *Francisella tularensis* live vaccine strain (LVS) digest were separated using the 2.1 × 150 mm separation column kept at 30 and 80 °C. The HTLC analyses were replicated using the trap-elute setup with the 2.1 × 5 mm Acquity UPLC Protein BEH C_4_ VanGuard Precolumn (shortly, BEH C_4_ trap column) maintained at 35 °C. Peptides were eluted using a linear gradient of 0.5–44.5% component B in 30 min. An isocratic hold at 0.5% B was run for 0, 30, 60, 120, and 240 min before gradient elution.

The LC–MS data were searched in Proteome Discoverer v2.3 using Byonic v3.5.0. After data recalibration, the spectra were searched against the *F. tularensis* subsp. *holarctica* protein database downloaded from UniProt. The mass tolerance was 7.5 ppm for precursors and 20 ppm for fragments. A semitryptic cleavage with two missed cleavages was used. Oxidized Met, pyroGlu formation from N-terminal Glu and Gln, and dehydrated Asp were set as dynamic modifications. Also, N-terminal S-carbamoylmethylCys cyclization was considered, and therefore, carbamidomethylation of Cys was set as a dynamic modification. The spectra acquired during the gradient elution were included in the data search. Peptide-spectrum matches identified with a 2D FDR ≤ 1% were considered.

The relative abundance of modified peptides was calculated as the number of peptides containing the modification divided by the number of peptides containing both the modified and unmodified amino acid. For N-terminal cyclization, the number of modified peptides was divided by the number of peptides with a corresponding N-terminal amino acid. Nonenzymatic hydrolysis at Asp was assessed as the number of peptides nontryptically cleaved at the C- or N-terminus of Asp divided by the total number of Asp-containing peptides.

### Artificial Modifications and Separation Performance in LC–MS Analyses with Various Gradient Times

Peptides from the Jurkat cells digest were separated using a 1 × 150 mm separation column kept at 30 and 80 °C. The high-temperature analyses were replicated using the trap-elute setup with a 1 × 5 mm EXP cartridge packed with BEH C_4_ particles maintained at 35 °C. The methods involved 30 and 60 min linear gradients of 0.5–42.5% component B and 120 and 240 min linear gradients of 0.5–39.5% component B. Two micrograms of peptides were separated in 30 min, 4 μg in 60 min, 10 μg in 120 min, and 20 μg in 240 min analyses. In addition, 50, 125, 300, 800, and 2000 ng of peptides were separated within 30 min using direct injection in the separation column maintained at 30, 60, and 80 °C and the trap-elute setup under the temperature regimes of 35/80 °C, 35/60 °C, and 22/60 °C. Eventually, the 60 min separations of 4 μg of peptides were replicated using direct injection in the separation column at 60 °C and the trap-elute setup with the 22/60 °C temperature setting.

The LC–MS data were processed analogously to the previous section using a human protein database and protein N-terminal acetylation as an extra variable modification.

### Quantity of Individual Modified Peptides and Separation Performance

Ten micrograms of digested trastuzumab were separated using a trap-elute setup consisting of a 2.1 × 5 mm BEH C_4_ trap column and a 2.1 × 150 mm separation column at the temperature regimes of 35/80 °C and 22/60 °C and direct injection in the separation column maintained at 30, 60, and 80 °C. The method included an isocratic hold at 0.5% component B for 1 min, followed by a linear increase to 29.5% B in 110 min.

The LC–MS data were searched against the FASTA sequence of trastuzumab using MSFragger integrated in Skyline v22.1 via the Peptide Search workflow.^26^ The cutoff score was set at 0.95. A semitryptic cleavage with two missed cleavages was used. Dehydration of Asp, oxidation of Met, pyroGlu formation from N-terminal Glu and Gln, and Asn deamidation were set as variable identifications. Possible precursor charges 2, 3, 4, and 5 were considered. The mass tolerance was 8 ppm for precursors and 18 ppm for fragments. Chromatograms from three monoisotopic peaks were extracted within 8 min around the identification time.

### Artificial Modifications in Peptide Mapping of Protein Biopharmaceuticals

For the final analysis of biopharmaceuticals, 10 μg of digested trastuzumab, aflibercept, bevacizumab, and durvalumab were separated using the 2.1 mm i.d. trap-elute setup at a temperature regime of 22/60 °C and direct injection in the 2.1 × 150 mm separation column maintained at 30 and 60 °C. The gradient was shortened to 90 min, and the final portion of the component B in the mobile phase increased to 33%. The LC–MS data were evaluated as described for trastuzumab above. Sequences of biopharmaceuticals were retrieved from DrugBank.^27^

## Results and Discussion

### Theoretical Considerations

The primary challenge in developing a trap-elute setup for minimizing the in-column peptide degradation in HTLC is to find a trap column that retains the peptides at a safe temperature for a substantial time but still enables their efficient refocusing at the head of the downstream separation column maintained at a high temperature. If the trap column retains peptides too much, then they elute with a strong mobile phase, which prevents them from maximum exploitation of the separation column efficiency. In the worst scenario, peptides pass through it without interacting with the stationary phase. This manifests in a significant increase in retention time and peak width at half height (*w*_h_). Under optimized retention in the trap column, the observed retention times should be shifted only by its dead time because the trap column contributes to the gradient delay time. However, even if the retention times are shifted only by the trap column dead time, the trap column can impair the observed separation, because it contributes to the extra-column band broadening.

Each chromatogram is a result of the in-column band broadening (σ_v, col_^2^) and the extra-column band broadening (σ_v, ec_^2^), determining the remaining column efficiency (*E*_r_) in %:
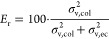
1

In gradient RPLC of peptides, only postcolumn band broadening (σ_v, post–col_^2^) contributes to extra-column broadening, while precolumn broadening is eliminated thanks to the refocusing of peptides injected in the initial weak mobile phase at the head of the separation column. However, if the refocusing is imperfect, the precolumn broadening contributes to the total band broadening by a factor determined by the retention in the mobile phase that delivers the peptides to the separation column, and the retention at the time of their elution from it.^28^ The retention in these two moments is determined by the retention factors *k*_0_ and *k*_e_, respectively:
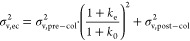
2

In the trap-elute setup, all parts upstream of the separation column in contact with injected peptides can contribute to precolumn band broadening, including the trap column itself. Assuming the trap column is retentive enough to refocus most peptides at its head, and the capillary connecting the trap column with the separation column is optimized, the precolumn broadening is largely determined by the broadening in the trap column:
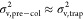
3

The band broadening in the trap column can be calculated identically to broadening in any column:
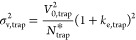
4

with *V*_0,trap_^2^ as the trap column dead volume, *N*_trap_^*^ as the plate number of the trap column in gradient separation, and *k*_e,trap_ as the retention factor of peptides when leaving the trap column (for further details, see a recent tutorial on RPLC of peptides^3^). Implementation of eq 4 into eq 2 subsequently highlights the parameters that determine the quality of peptide separation in the trap-elute setup:
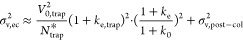
5

The band broadening downstream of the separation column is usually well optimized, and it is the same in the direct injection as that in the trap-elute setup. Therefore, further attempts to reduce it do not improve the observed separation in the trap-elute setup compared to the direct injection.

Eq 5 implies that for superior separation performance of peptides in the trap-elute setup, peptides must have significantly larger *k*_0_ than *k*_e_. For instance, if the trap column allows the peptides to arrive in the separation column with *k*_0_ 5.4-fold greater than *k*_e_, then the overall effect of the first term in eq 5 is reduced by more than 90%. This can be achieved particularly by using a lower retentivity sorbent in the trap column. Another means for improvements involves using highly efficient trap columns that provide a high plate number and decreased dead volume, for instance, by packing them with superficially porous particles with a thin, porous layer. Band dispersion in the trap column can be reduced by decreasing the retention factor of peptides when leaving the trap column by increasing its length or inner diameter. However, increasing these dimensions could result in the peptides eluting from the trap column with a stronger solvent, thereby decreasing their *k*_0_ in the separation column. This explains why the trap column must be short and its inner diameter should not exceed the inner diameter of the separation column. Eq 5 also explains that the trap-elute setup is easier optimized for longer gradients realized using longer separation columns because both these parameters decrease *k*_e_.

An increment of 5 °C has an effect analogous to increasing acetonitrile concentration in the mobile phase by 0.3–1%.^8^ Therefore, adjusting the temperature can help fine-tune the retentivity of both columns in the trap-elute setup. However, the effects of the temperature on the separation performance and in-column degradation of peptides should be kept in mind.

An additional consideration in the trap-elute setup with different temperatures must be given to the capillary connecting the trap and separation column, where the balance between the band dispersion inside it and temperature mismatch must be found. The band dispersion due to the laminar flow in capillaries (σ_v, tub_^2^) can be estimated from^29^
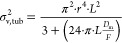
6

The equation implies that the major impact on the dispersion in tubing possesses the inner radius of the capillary (*r*), while its length (*L*) and flow rate (*F*) are less important. If the capillary is placed in the column thermostat, the impact of a lower diffusion coefficient (*D*_m_) of peptides is not pronounced much because it rises with temperature.

While minimum capillary dimensions are needed to limit the band dispersion, this can lead to temperature mismatch. This phenomenon is caused by the poorly preheated mobile phase that cools the separation column, creating a radial temperature gradient. The axial temperature heterogeneity leads to gradients of viscosity and strength of the mobile phase, resulting in peak distortions.^30−32^ The required length of a capillary to be heated to avoid the temperature mismatch can be estimated from
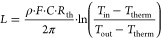
7

with the parameters of mobile phase (ρ density, *C* heat capacity), capillary resistance to heat transfer (*R*_th_), the temperature of the mobile phase entering and leaving the capillary (*T*_in_ and *T*_out_, respectively), and thermostat temperature (*T*_therm_).^33^ The equation implies that the length must correspond to the column temperature and flow rate. Conversely, if the capillary wall is thin and is made of a material with high thermal conductivity, such as stainless steel, the preheating efficiency of the capillary is hardly affected by its inner diameter.^32^ As a result, the length rather than the inner diameter of the heated capillary between the trap and separation column should be increased to find an optimal balance between the band dispersion and temperature mismatch. The largest minimum length of the capillary predicted using eq 7 needed to avoid temperature mismatch was 18 cm (Table S3).

### Trap-Elute Setup Optimization

#### Separation Column

In this study, we exclusively used separation columns packed with a particulate C_18_ stationary phase that featured a positively charged surface. This sorbent was specifically developed to facilitate the separation of protonated analytes, including peptides, using an MS-friendly mobile phase.^34^ In our experience, the marketed Charged Surface Hybrid (CSH) stationary phase offers superior peptide separation.^4,35^ However, this improvement comes at the expense of reduced retentivity, as the CSH C_18_ stationary phase is among the least retentive sorbents with octadecyl ligands.^34,36,37^ Consequently, optimizing the use of CSH C_18_ columns in the trap-elute setup poses challenges. A slight increase in the retentivity of the CSH C_18_ stationary phase compared to its noncharged counterpart can be achieved by decreasing the formic acid concentration, while the effect on a regular C_18_ stationary phase is opposite.^35^ For this reason, we acidified both components of the mobile phase with 0.05% formic acid. We trust that optimizing a trap-elute setup with more retentive separation columns will be easier because of a wider range of compatible trap columns and a larger space for tuning the column temperatures.

#### Trap Column

**Figure 1:**
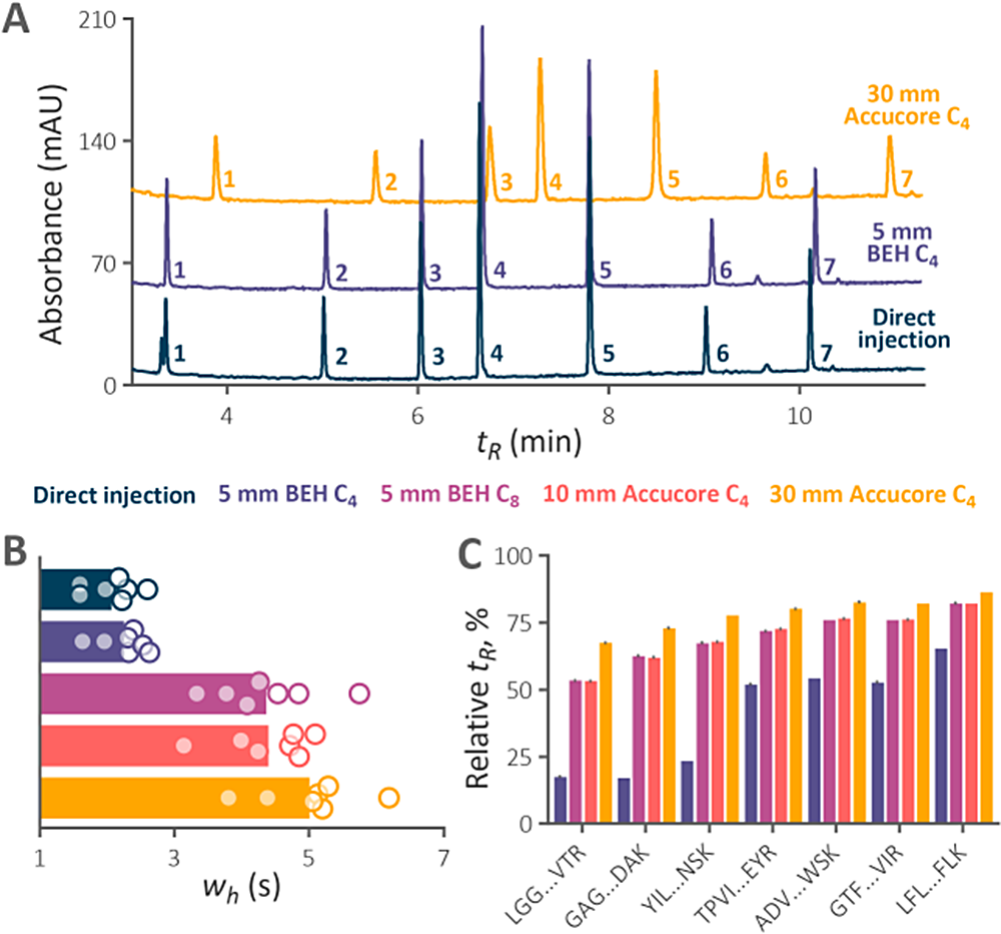
Selection of the trap column. (A) Overlaid chromatograms of the 10 min gradient separation of iRT peptides using direct injection in the 2.1 mm inner diameter separation column and the worst and best performing trap-elute setups. The numbers near the peaks correspond to the used iRT peptides. (B) Average peak width (*w*_h_) of iRT peptides obtained using direct injection in the 2.1 mm inner diameter separation column and using trap-elute setups. The averaged *w*_h_ values for each peptide from the 30 min separation are shown. (C) Relative portion of the total retention time (*t*_R_) that iRT peptides spent in the 2.1 mm i.d. trap columns during the 30 min separation, calculated as *t*_R_ of peptides in the trap columns divided by *t*_R_ in the trap-elute setups. The separation and trap columns were maintained at 70 and 22 °C, respectively.

The study began with searching for the optimal 2.1 mm inner diameter trap column. While all examined trap columns provided significantly weaker retention than the separation column during isocratic elution of small analytes (Figure S1), most of them notably broadened the peaks during gradient elution of iRT peptides (Figure 1). Although the peptides spent a major portion of the total retention time in the trap columns, their strong retention hindered efficient refocusing in the separation column. Consequently, upstream band broadening was not eliminated, leading to a decrease in the overall separation performance. The only exception was a 2.1 × 5 mm BEH C_4_ trap column, which retained most peptides for more than half of the total retention time while broadening their peaks only by 6.5%. The portion of the relative retention time that peptides spent in the trap column increased with the peptide hydrophobicity and the gradient length. Hydrophilic peptides were retained in the trap column only for approximately 20% of the total retention time. However, since these peptides also spend less time in the separation column, the risk of their degradation is minimal. Consequently, the 2.1 × 5 mm BEH C_4_ column was selected for the 2.1 mm i.d. trap-elute setup.

For the 1 mm inner diameter separation column, none of the commercially available 1 × 5 mm EXP Trap cartridges preserved the intrinsic performance of the separation column. Eventually, the EXP Trap cartridge packed on demand by the hardware manufacturer with the BEH C_4_ particles provided satisfying results analogously to the 2.1 mm i.d. setup.

In the LC–MS experiments, the separation column temperature was 80 °C. Since higher temperatures reduce retention, we decreased *k*_e,trap_ by thermostatting the trap column at 35 °C to ensure that peptides reached the separation column with a weak mobile phase for efficient refocusing. This temperature was chosen as a balance between minimizing the level of peptide modification in the trap column and preserving the separation efficiency.

#### Connecting Capillary

The dimensions of the capillary connecting the trap and separation column had a minor effect on the quality of iRT peptides separation. The difference in the average *w*_h_ between the best and the worst performing capillaries was below 3% in the 30 min gradient separation, except for the 0.075 × 150 mm PEEK-shielded fused silica nanoViper capillary in the 2.1 mm i.d. setup, which broadened peaks by 5.7% (Figure S2). The less thermally conductive material of that capillary and its length likely prevented sufficient preheating, resulting in a temperature mismatch. The passage time did not significantly correlate with the observed *w*_h_. The nanoViper capillaries provided the worst peaks also in the 1 mm i.d. setup, although the flow rate decreased proportionally to 68 μL/min, suggesting that the material was more important for preheating the mobile phase than the inner diameter in our settings.^32^

As even the largest capillary did not worsen the separation, we concluded that refocusing at the head of the separation column eliminated the upstream peak broadening. This secondarily proved the proper selection of parameters of the trap column, which enabled the peptides to reach the separation column in a sufficiently weak mobile phase. Based on the results, we used the 0.1 × 380 mm stainless steel capillary in the following experiments because it enabled active preheating and was long enough to connect both columns in two separate thermostats.

### Artificial Modifications in Model LC–MS Analyses with Various Isocratic Hold Times

**Figure 2:**
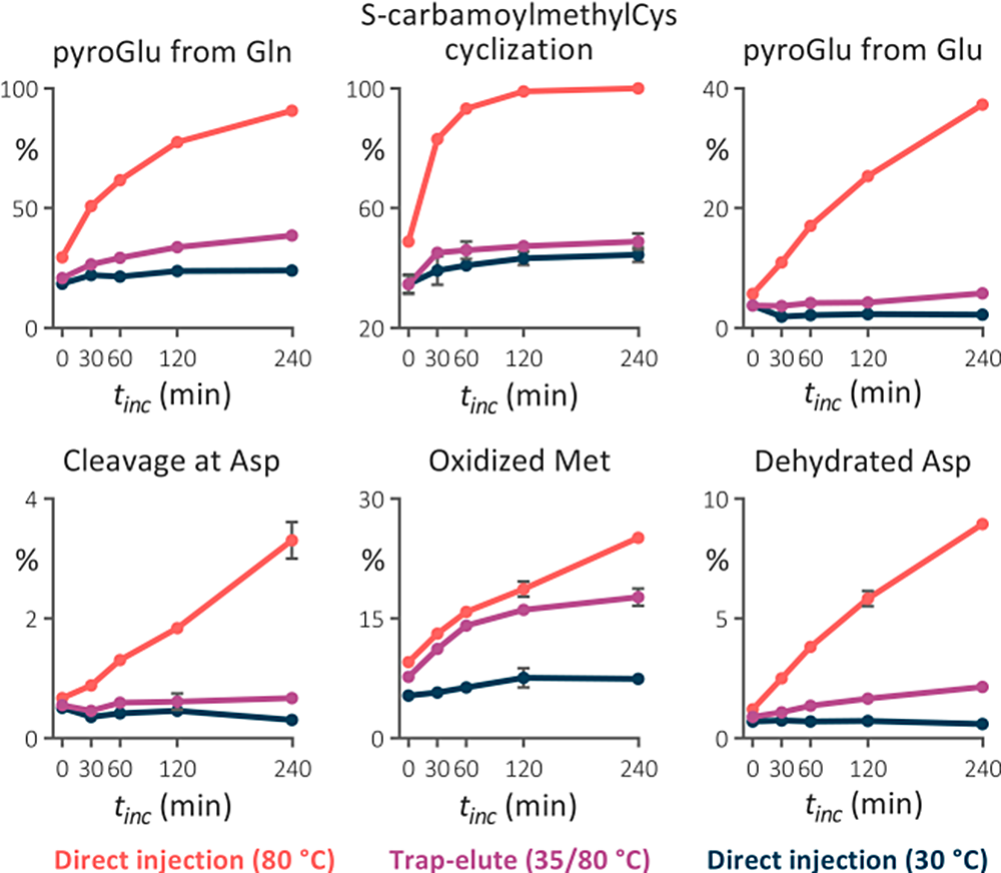
Relative abundance of temperature-related artifacts identified in the tryptic digest of *F. tularensis* LVS in-column incubated for various times (*t*_inc_). Analyses were performed using direct injection in the 2.1 × 150 mm separation column maintained at 30 and 80 °C and the trap-elute setup in the temperature regime of 35/80 °C. Very small error bars are covered by the data symbols.

To demonstrate the efficiency of the trap column in reducing artificial modifications in HTLC–MS of peptides, we conducted an experiment similar to that previously employed.^4^ The isocratic hold before the gradient elution extended the in-column residence time of peptides, simulating conditions encountered during long gradient analyses. In accordance with the previous findings, the abundance of in-column generated modifications increased with both the column temperature and the in-column residence time of peptides (Figure 2). For the first time, we revealed that cyclization of N-terminal carbamidomethylated Cys can also proceed in the column. The analysis conducted at 80 °C served as a reference when no preventative measures were applied. The abundance of modifications generated at 30 °C was considered the lowest achievable, and since it did not increase with the in-column time, they must have been generated prior to the analysis.

Reassuringly, the installation of the inline trap column reduced the abundance of all traced modifications, with its benefits becoming more pronounced during longer incubation times, suggesting its particular advantage for extended analyses. For Asp dehydration, nonenzymatic cleavage at Asp, carbamoylmethylCys cyclization, and pyroGlu formation, the modification extent almost reached the background level observed at 30 °C. Partial improvement was also observed in Met oxidation. The trap column retained around 75% of the peptides that were efficiently retained at the head of the separation column (Figure S3). The untrapped hydrophilic peptides quickly exited the separation column, so they were not excessively exposed to high temperatures. The number of peptides identified in subsequent blank injections did not exceed those in blanks after the direct injection, indicating no increase in sample carryover due to the trap column being kept at a low temperature.

### Artificial Modifications and Separation Performance in LC–MS Analyses with Various Gradient Times

**Figure 3:**
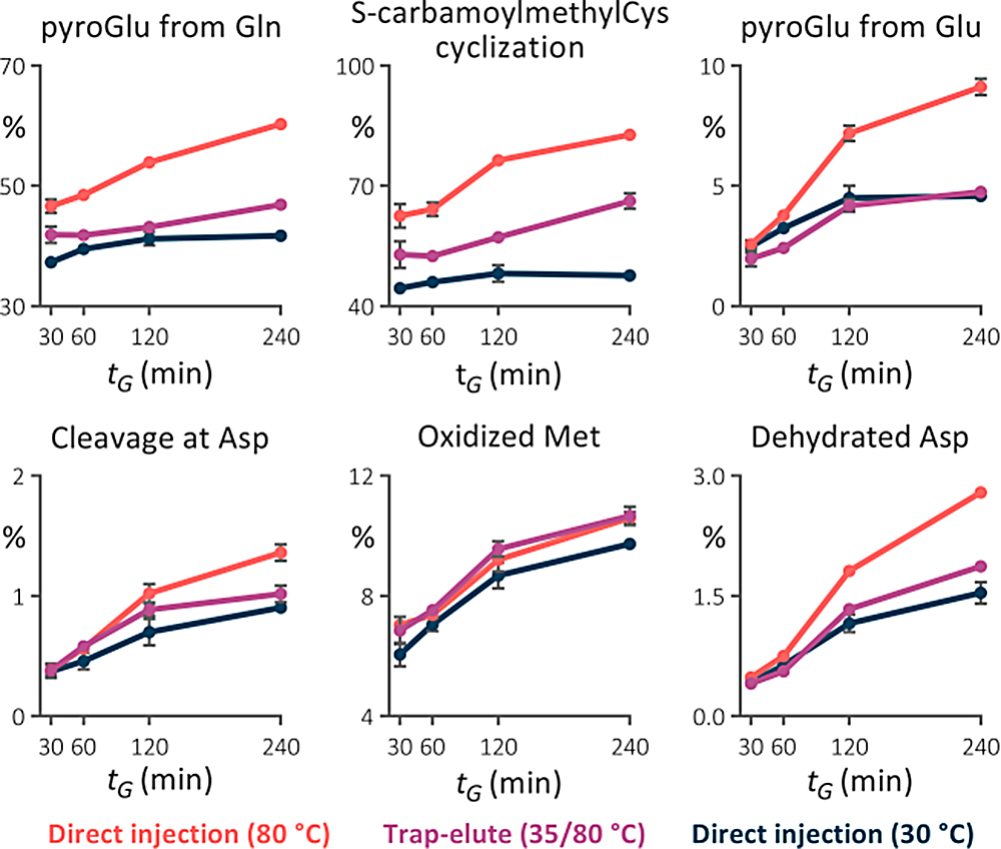
Relative abundance of temperature-related artifacts identified in analyses of the Jurkat cells digest performed using the 1 mm inner diameter trap-elute setup in the temperature regime of 35/80 °C and direct injection in the 1 × 150 mm separation column maintained at 30 and 80 °C. Four different active gradients were used. Very small error bars are covered by the data symbols.

We assessed data from real-life microflow LC–MS bottom-up proteomic analyses to confirm the benefit of the trap-elute setup on the abundance of in-column generated artifacts observed in the model LC–MS analyses. The trap-elute setup successfully reduced Asp dehydration, pyroGlu formation, carbamoylmethylCys cyclization, and Asp-specific nonenzymatic cleavage (Figure 3). Predictably, the benefit was smaller than in the model experiment, where peptides spent substantially more time in the trap column than under gradient elution in real-life analyses. Nevertheless, the trap column still prevented the modification of at least 50% of peptides containing Asp and N-terminal Glu or Gln that otherwise would have been modified. The installation of the trap column was increasingly beneficial in longer gradient runs for all of the responsive modifications.

No improvement in Met oxidation was observed compared with the model analyses. In addition, the portion of peptides containing oxidized Met did not increase with the column temperature. Since the 1 mm i.d. separation column lacked coating that reduces exposure to the metal surface,^38^ unlike the 2.1 mm i.d. column used in the previous experiment, residual metals likely played the main role in Met oxidation, while the contribution of the column temperature was minor. Consequently, the opportunity to affect this modification by the trap column was greatly diminished.

**Figure 4:**
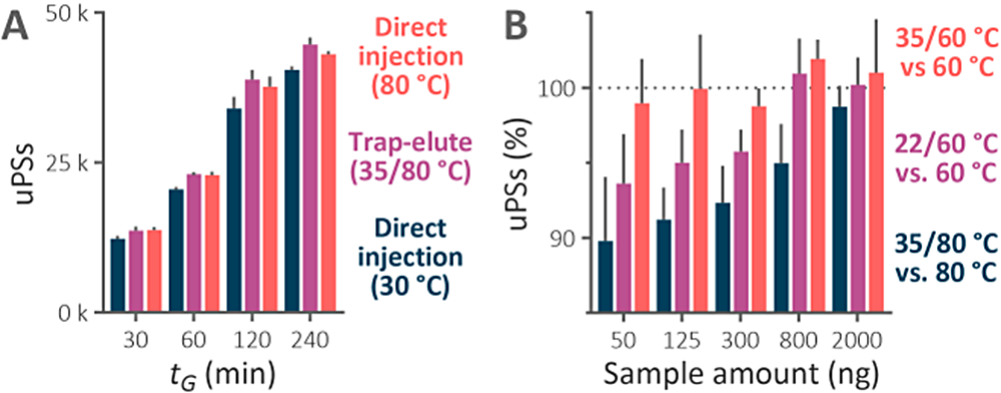
(A) Number of unique peptide sequences (uPSs) identified in analyses of the Jurkat cells digest using the 1 mm inner diameter trap-elute setup in the temperature regime of 35/80 °C and direct injection in the 1 × 150 mm column held at 30 and 80 °C. (B) Relative quantity of uPSs identified in the 30 min analyses of reduced masses of the Jurkat cells digest using three temperature settings of the trap-elute setup. The identified uPSs using the trap-elute setup were normalized to the identifications obtained from analyses using direct injection in the separation column maintained at the corresponding temperature.

The increased number of identified peptides represents the key benefit of HTLC that results from improved RPLC separation.^4^ We evaluated the performance of the trap-elute setup in terms of identified peptides using unique peptide sequences (uPSs) regardless of the presence of artificial modifications. Predictably, more uPSs were identified using the separation column maintained at 80 °C compared to 30 °C for all tested gradient lengths (Figure 4A). However, the relative increase in the number of uPSs declined from 10.9% in the 120 min analysis to 6.5% in the 240 min analysis, arguably because of artificial modifications. The trap-elute setup provided an identical number of identified uPSs in 30 and 60 min analyses as in the HTLC analysis using direct injection. Crucially, for 120 and 240 min analyses, the trap column increased the number of uPSs compared to the corresponding analyses using the separation column at 80 °C by 3.1 and 3.6%, respectively. The trap-elute setup in the longer analyses very likely protected the unmodified parent peptides by reducing their degradation; therefore, their intense precursors were more often selected for fragmentation. The trap column also reduced the number of redundant peptide sequences that needlessly competed for sequencing with unique peptides (Figure S4A). This likely further contributed to the increased number of identified uPSs in 120 and 240 min analyses with the trap column. Additionally, the trap column reduced the number of peptides identified only in the modified form (Figure S4B).

The experiment was carried out using a higher mass of peptides to obtain a statistically relevant amount of data. However, because of the relationship between the amount of injected sample and the number of identified peptides typical for saturation binding experiments,^3^ such a sample amount could mask the impact of potentially decreased separation performance on the number of uPSs due to installing the trap column. Therefore, we also injected smaller peptide masses. Naturally, identifications decreased with reduced sample amounts, but the decrease was more pronounced in the trap-elute setup. For the smallest peptide mass, the difference in uPSs between direct injection and the trap-elute setup reached 10.2% (Figure 4B). Fewer identifications correlated with an increased average *w*_h_ (Figure S5).

Compared to direct injection, fewer uPSs were identified from the reduced masses of peptides in the trap-elute setup under the temperature regime of 35/80 °C, and the trap column did not reduce the modifications to the minimum observed level at 30 °C. We hypothesized that both limitations could be addressed by lowering the temperature of the separation column. Its higher retentivity at lower temperatures should mitigate peak broadening associated with the trap column. Moreover, if the retentivity was high enough, it would likely enable using the trap column at ambient temperature with no need for an additional thermostat in an air-conditioned laboratory. Finally, as the separation column temperature primarily determines the extent of modification, its reduction should further decrease their abundance. Therefore, we decreased the temperatures of both columns.

First, we decreased the temperature of the trap column from 35 °C to ambient 22 °C. Using iRT peptides, we determined that the highest temperature of the separation column that enabled the use of the trap column at 22 °C without noticeable peak broadening was 60 °C. In this adjusted regime of 22/60 °C, the peaks of iRT peptides were broadened only by 1.0% after installing the trap column. Subsequently, we compared the temperature regime of 22/60 °C to that of direct injection at 60 °C and collated the results with those obtained from the 35/80 °C regime. When the injected sample amount was reduced, the new setting significantly decreased the relative gap in identified uPSs between direct injection at 60 °C compared to 35/80 °C vs 80 °C (Figure 4B). A constant number of uPSs was identified with and without the trap column from 2 μg of the sample. Their number was similar not only within the data sets of 60 °C with and without the trap column but also in comparison to the analogous data sets of 80 and 35/80 °C. Hence, decreasing the separation column temperature did not reduce the method performance in terms of identified uPSs. More importantly, it minimized peak broadening due to trap column installation (Figure S5), which impaired analyses of very small sample amounts (Figure 4B). These drops of identified uPSs were avoided when the trap column temperature was elevated back to 35 °C.

Users can decide whether to increase the trap column temperature or keep it ambient for particular analyses. The trap column held at 35 °C maintained superior performance even for small sample amounts at the level provided by direct injection in the separation column kept at 60 °C. By maintaining the trap column at ambient temperature, users must tolerate a moderate drop in identifications when analyzing smaller input masses. However, even with the trap column kept at ambient temperature, fewer identifications observed in the analysis of 50 ng of peptides are still far above the number obtained using direct injection at 30 °C (−6.4% vs −24.5%). That is why we considered this drop to be negligible.

The abundance of modified peptides at 60 °C decreased already without the trap column, but a modification level close to that typical for the analyses at 30 °C was reached only with the trap column (Figure S6). This experiment also confirmed that modifications occur already at a moderately elevated column temperature of 60 °C, although their abundance is significantly lower than that at 80 °C.

### Quantity of Individual Modified Peptides and Separation Performance

To investigate the extent of artificial modifications at the level of individual peptides, we quantified specific artifacts within the structure of a model protein. We selected trastuzumab for this investigation to leverage its exceptional purity and the appropriate quantity of predicted tryptic peptides.

**Figure 5:**
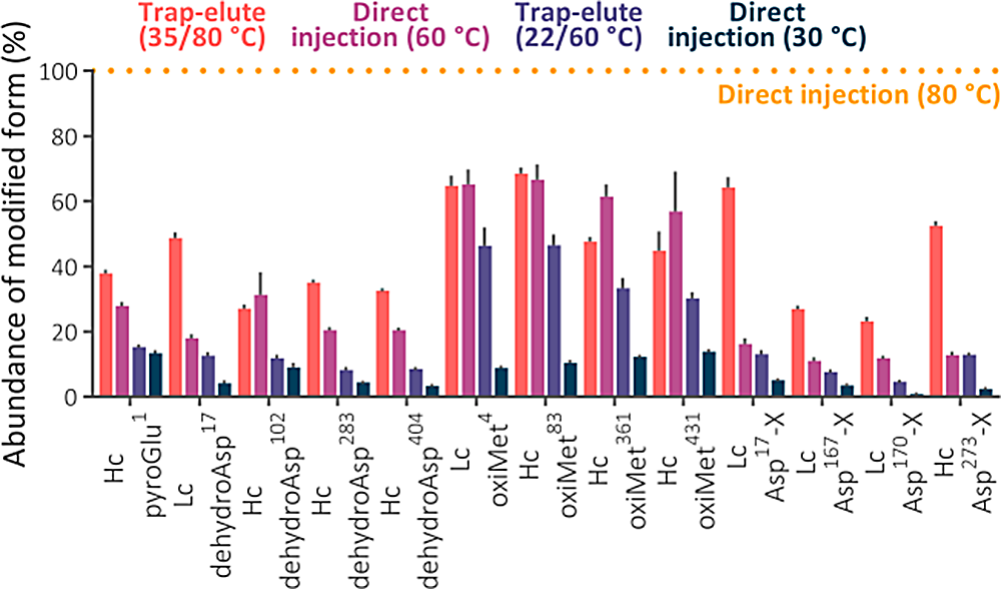
Abundance of the modified peptide forms in the 110 min analyses of trastuzumab using temperature regimes of 22/60 and 35/80 °C and direct injection in the separation column maintained at 30 and 60 °C. The abundance was calculated as the peak area of the modified peptide divided by the summed area of both peptide forms. The abundance of the modified form was then normalized to that observed in the analyses using the direct injection in the separation column maintained at 80 °C (100%, dotted line). The peak area was calculated from the most intense precursor charge state. The most abundant modified peptide containing the modified amino acid was used.

**Figure 6:**
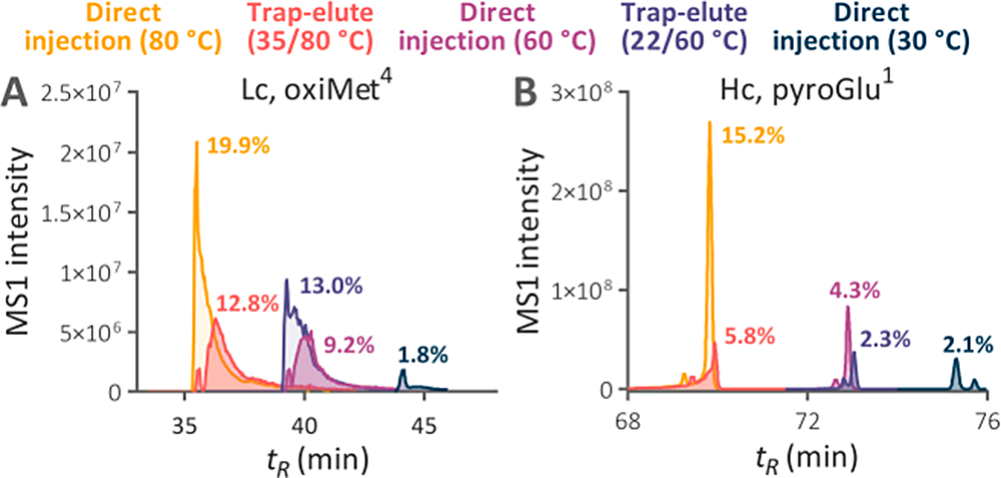
Extracted ion chromatograms of peptides (A) D^1^IQM[oxi]TQSPSSLSASVGDR^18^ and (B) E^1^[pyro]VQLVESGGGLVQPGGSLR^19^ from trastuzumab digest analyzed within a 110 min gradient using the temperature regimes of 22/60 and 35/80 °C and the direct injection in the separation column maintained at 30, 60, and 80 °C. The % values represent the modified peptide peak area divided by the summed peak area of both peptide forms.

The trap-elute setup at the temperature regime of 22/60 °C reduced the quantity of most traced modified peptides almost to the levels observed using direct injection at 30 °C (Figure 5), decreasing their abundances below 2% (Figure S7). In contrast to the analyses of the Jurkat cells digest using an uncoated 1 mm inner diameter separation column, we observed improvement in the oxidation level of individual Met residues in trastuzumab (Figures 5 and 6A) in the 2.1 mm inner diameter trap-elute setup. This confirmed that the column temperature has a major effect on this modification when the hybrid surface technology prevents the contact of peptides with the metal hardware.^38^ For other modifications, their decreased quantities correlated with the previously observed decrease in the number of identified modified peptides.

Due to the benefits of HTLC separation, the peak capacity was more than 1.4-fold higher at the temperature regime of 22/60 °C compared to the separation at 30 °C. The peak capacity was almost identical for the 60 °C analyses with and without the trap column (776 vs 786), confirming that the intrinsic performance of the separation column was almost completely preserved (Figure S8). LC–MS analyses using direct injection at 80 °C provided a superior peak capacity of 842, but it decreased to 802 with a trap column maintained at 35 °C.

### Artificial Modifications in Peptide Mapping of Protein Biopharmaceuticals

The in-column generated modifications are of particular importance in the quality control of protein biopharmaceuticals because they can falsely increase the levels of modifications monitored as CQA. We observed such an example for N-terminal pyroGlu of the trastuzumab heavy chain in the previous analyses (Figure 6B).^39^

Based on these findings, we hypothesized that the in-column artifacts could distort the quantification of certain CQAs in several previous studies exploiting HTLC methods.^40−44^ Whereas the impact of artificial modifications generated during sample preparation for quality control of protein biopharmaceuticals is well-known,^45,46^ the concerns about the impact of in-column artifacts are discussed in the literature very sporadically. Most of them are focused on Met oxidation.^11−13^ Therefore, we evaluated 33 publications available on the Web of Science that were published in 2022–2023 and involved the characterization of therapeutic proteins using peptide mapping. Among the 27 studies with specified column temperatures, ≥45 °C was used in 22 of them and a temperature of ≥60 °C was used in 10 (Table S4). An average gradient length exceeded 1 h. This indicates that the impact of the in-column generated artifacts on the quantification of modifications concerns many laboratories and needs to be addressed. Adjustments can involve shortening the method and decreasing the column temperature. However, these adjustments will negatively affect the separation and likely will not lead to the complete elimination of the artifacts. Moreover, they can dramatically change some parameters monitored for method validation.

We suggest that implementing the proposed trap-elute setup might substantially improve the reliability of the analysis of CQA. Although the 35/80 °C setting provides the best separation performance and may be superior for peptides problematic for RPLC,^4^ it might not suffice for achieving the utmost reduction in artificial modifications. The preferred temperature regimes in this niche application are 22/60 and 35/60 °C. Compared to shortening the method and decreasing the column temperature, our approach barely changes chromatograms and requires no adjustment of the LC program (Figure S9).

To emphasize the importance of in-column generated modifications in HTLC of protein biopharmaceuticals and demonstrate how the trap-elute setup can reduce them, we mapped peptides of trastuzumab, bevacizumab, aflibercept, and durvalumab using typical method parameters from recent relevant studies, i.e., column temperature of 60 °C and gradient time of 90 min. The peptides were analyzed using direct injection at 30 and 60 °C and the trap-elute setup in the temperature regime 22/60 °C. The results were in line with the initial peptide mapping of trastuzumab (Figure S10). Compared to direct injection at 60 °C, the trap-elute setup generated on average 65.7 ± 11.2% less temperature-related artifacts with N-terminal pyroGlu, 62.9 ± 23.6% of those with oxidized Met, and 71.5 ± 14.8% of those with dehydrated Asp. This confirmed the efficiency of the trap-elute setup in coping with the in-column generated artifacts in this niche application.

## Conclusions

Following our characterization of the in-column generated modifications during HTLC of peptides,^4^ we aimed to devise a method that could fully retain the benefits of high column temperature for peptide separation while minimizing in-column generated modifications. In a recent study, we demonstrated that decreasing the acidity of the mobile phase did not yield the desired effect.^35^ Consequently, we focused on the in-column residence time as another aspect associated with the artificial modification of peptides in HTLC. Compared to the analyses at 30 °C, our simple approach relying on a trap column provided a 1.4-fold higher peak capacity in 110 min analyses of biopharmaceuticals and identified 10% more uPSs in bottom-up proteomic analyses while maintaining most modifications close to the minimum level. In the tailored temperature regime of 35/60 °C, the trap-elute setup preserved the number of identified peptides even for small sample loads. We demonstrated that the trap column can be maintained even at ambient temperature, eliminating the need for an additional thermostat. After thoroughly optimizing and evaluating the concept in bottom-up proteomics, we implemented it in the peptide mapping of protein biopharmaceuticals. Our method reduced the in-column artificial modification without causing other significant changes in chromatograms. The concept is easy to implement in existing methods, which is technically equivalent to adopting a guard column. Indeed, the inline trap column can also serve as a guard column, extending the lifespan of the separation column.

The method is especially advantageous in applications with limited tolerance to artificial modifications, such as multiattribute methods for quality control of protein biopharmaceuticals. We believe that our approach will make HTLC more appealing to practitioners seeking straightforward solutions to improve chromatographic performance across various applications and not just in the analysis of peptides and proteins.

## Supporting Information Available

Trap-elute setup configuration (Scheme S1); sample preparation (Note S1); ion source settings (Table S1); settings of DDA experiments (Table S2); predicted minimum lengths of the capillary connecting the trap and separation column to avoid temperature mismatch (Table S3); recent publications involving peptide mapping of biopharmaceuticals (Table S4); isocratic separations of small organic analytes (Figure S1); comparison of connecting capillaries (Figure S2); portion of peptides effectively retained in the trap column upon injection (Figure S3); redundant peptide sequences and peptides identified only with artificial modifications (Figure S4); peak broadening due to the trap column installation (Figure S5); abundance of artifacts in analyses using the optimized temperature setting (Figure S6); significance of artificial modification for peptides of monoclonal antibody (Figure S7); peak capacity and peak width distribution in model analyses of trastuzumab (Figure S8); illustrative chromatograms with and without the trap column (Figure S9); and artificial modifications in four protein biopharmaceuticals (Figure S10) (PDF)
