# Supplementary material for: Mitigating In-Column Artificial Modifications in High-Temperature LC–MS for Bottom–Up Proteomics and Quality Control of Protein Biopharmaceuticals
Source: Anal Chem. 2024 Aug 28;96(36):14531–40. doi: 10.1021/acs.analchem.4c02819 (PMC11391404; doi:10.1021/acs.analchem.4c02819)
Supplement: Supplementary file 1 — ac4c02819_si_001.pdf [file ac4c02819_si_001.pdf]

Supporting Information

Mitigating in-column artificial modifications in high-temperature LC-MS for bottom-up proteomics and quality control of protein biopharmaceuticals

Mykyta R. Starovoit, Siddharth Jadeja, Taťána Gazárková, Juraj Lenčo*

Department of Analytical Chemistry, Faculty of Pharmacy in Hradec Králové, Charles University, Heyrovského 1203/8, 500 03 Hradec Králové, Czech Republic

*Corresponding Author: E-mail: lenco@faf.cuni.cz, Phone: +420 495 067 381

Table of Contents

Scheme S1: Trap-elute setup configuration S2

Note S1: Sample preparation S3

Table S1: Ion source settings S4

Table S2: Settings of DDA experiments S5

Table S3: Predicted minimum lengths of the capillary connecting the trap and separation column to avoid temperature mismatch S7

Table S4: Recent publications involving peptide mapping of biopharmaceuticals S8

Figure S1: Isocratic separation of small organic analytes S11

Figure S2: Comparison of connecting capillaries S12

Figure S3: Portion of peptides effectively retained in the trap column upon injection S13

Figure S4: Peptides identified only as modified and sequence duplicates S14

Figure S5: Peak broadening after the trap column installation S15

Figure S6: Abundance of artifacts in analyses using the optimized temperature setting S16

Figure S7: Significance of in-column artificial modification for peptides of monoclonal antibody S17

Figure S8: Peak capacity and peak width distribution in model analyses of trastuzumab S18

Figure S9: Illustrative chromatograms with and without the trap column S19

Figure S10: Artificial modifications in four protein biopharmaceuticals S20

Supporting References S21

Scheme S1: Trap-elute setup configuration

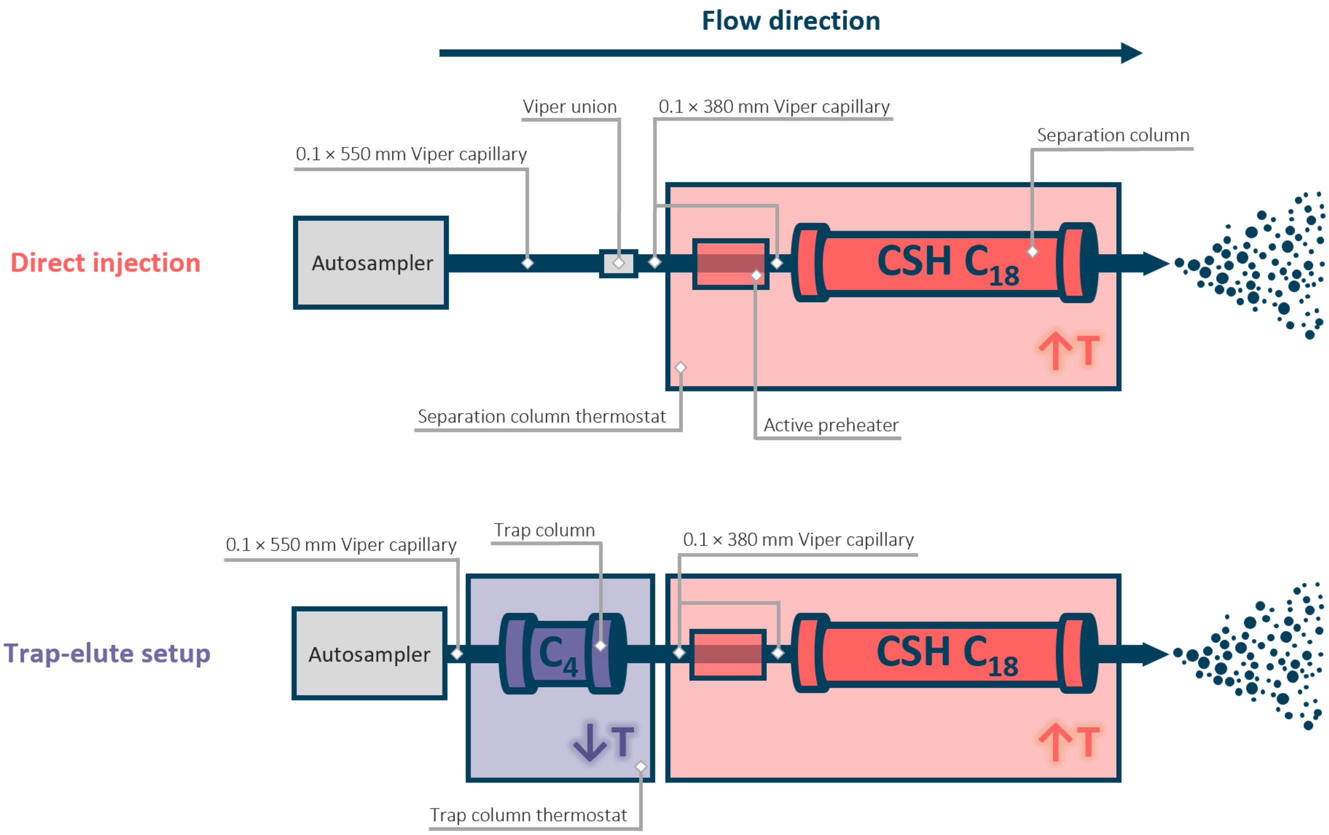

The LC-MS instrument configuration of the direct injection and trap-elute setup. As VanGuard precolumns have a male-threaded outlet, they were connected to the 0.1 × 380 mm Viper capillary through a zero-dead-volume Viper union (not shown).

Note S1: Sample preparation

iRT peptides

Stock solutions of seven iRT peptides [1] (LGGNEQVTR, GAGSSEPVTGLDAK, YILAGVENSK, TPVITGAPYEYR, ADVTPADFSEWSK, GTFIIDPGGVIR, LFLQFGAQGSPFLK) were mixed so that the concentration of each was around 0.125 µg/µL. The mixture was 4.4-fold diluted for microflow analyses. The sample contained 20% acetonitrile and 0.001% PEG 20 000.[2] The aliquots of the mixture were stored at -20 °C.

Trypsin digestion of proteins of *Francisella tularensis* and Jurkat cells

The *F. tularensis* live vaccine strain (LVS) was obtained from Chamberlain medium culture with OD_{600} of 0.6 to 0.7. The pellet was lysed in 2% sodium deoxycholate at 70 °C for 5 min. The proteins were incubated with 250 UI of benzonase for 30 min at 37 °C in the presence of 100 mM Tris-HCl buffer, pH 7.5.

The Jurkat cells (ATCC TIB-152) were cultured in 150 cm^2 cultivation flasks (TPP) in an RPMI 1640 medium supplemented with 10% fetal bovine serum. The cells were washed with phosphate-buffered saline and lysed on ice with 2.5% sodium deoxycholate containing 125 UI/mL benzonase. The protein concentrations were determined using a bicinchoninic acid assay (Merck/Sigma Aldrich).[3]

One milligram of the proteins from both lysates was subjected to reduction of disulfides in 5 mM TCEP for 60 min at 37 °C. Free thiols were blocked in 15 mM iodoacetamide at room temperature within 30 min in the dark. Iodoacetamide was quenched by incubation in 20 mM L-cysteine. The mixtures were then diluted with 50 mM Tris-HCl buffer to reach a pH above 7. The proteins were digested using trypsin in a 1:50 enzyme-protein ratio at 37 °C overnight. The digests were acidified with trifluoroacetic acid (TFA) to quench the enzymatic reaction and induce precipitation of deoxycholic acid, which was subsequently extracted into ethyl acetate saturated with water.[4] The remnants of ethyl acetate were evaporated in a vacuum centrifuge at 30 °C within 30 min. The peptides were desalted using a Pierce Peptide Desalting Spin Column (Thermo Fisher Scientific) according to the manufacturer's manual. The eluates were vacuum-dried. The dried peptides were dissolved in 0.1% aqueous TFA with 0.001% PEG 20 000 and stored at -80 °C till analysis.[2,5]

Trypsin digestion of protein biopharmaceuticals

Biopharmaceuticals were denatured in 5 M guanidinium chloride and reduced in 20 mM DTT for 30 min at room temperature. Thiols were not blocked. [6-8] The denatured biopharmaceuticals were diluted with the Low-Artifact Digestion Buffer containing DTT so that its concentration was 2 mM. The proteins were digested by trypsin added in a 1:20 enzyme-protein ratio at 37 °C for 2 hours. The digests were acidified with TFA, supplemented with 0.001% PEG 20 000, and stored at -80 °C till analysis.[2,5]

Table S1: Ion source settings

	1 mm i.d. columns	2.1 mm i.d. columns
Mobile phase flow rate	50 µL/min	300 µL/min
**Ion Source Parameter**		
Sheath gas flow rate	30.0	48.0
Aux gas flow rate	10.0	11.0
Sweep gas flow rate	1.0	2.0
Spray voltage, kV	3.5	3.5
Capillary temperature, °C	250.0	256.0
Aux gas heater temperature, °C	150.0	413.0
Depth of the ESI probe	A	halfway between B-C

Table S2: Settings of DDA experiments

Artificial modifications in model LC-MS analyses with various isocratic hold times	
**MS1 settings**	
Resolution at 200 m/z	60 000
Automatic gain control target	3×10^6
Maximum injection time	110 ms
Scan range	350 to 1500 m/z
**DDA and MS2 settings**	
Max. number of precursors	10
Precursor charge states	2, 3, 4, 5
Intensity threshold	1×10^5
Isolation window	1.8 m/z
Isolation offset	0.3 m/z
Normalized collision energy	27
Resolution at 200 m/z	15 000
Automatic gain control target	2×10^5
Maximum injection time	50 ms
Exclusion of isotopes	On
Dynamic exclusion time	20.0 s

Artificial modifications and separation performance in LC-MS analyses with various gradient times	
**MS1 settings**	
Resolution at 200 m/z	60 000
Automatic gain control target	3×10^6
Maximum injection time	110 ms
Scan range	350 to 1500 m/z
**DDA and MS2 settings**	
Max. number of precursors	15
Precursor charge states	2, 3, 4, 5
Intensity threshold	1×10^5
Isolation window	1.8 m/z
Isolation offset	0.3 m/z
Normalized collision energy	27
Resolution at 200 m/z	15 000
Automatic gain control target	2×10^5
Maximum injection time	50 ms, 75 ms [a]
Exclusion of isotopes	On
Dynamic exclusion time	15.0 s, 30.0 s, 60.0 s, 120.0 s [b]

**MS1 settings**	
Resolution at 200 m/z	60 000
Automatic gain control target	1×10^6
Maximum injection time	118 ms
Scan range	250 to 1500 m/z
**DDA and MS2 settings**	
Max. number of precursors	3
Precursor charge states	2, 3, 4, 5
Intensity threshold	2.5×10^5
Isolation window	2.5 m/z
Isolation offset	0.3 m/z
Normalized collision energy	27
Resolution at 200 m/z	30 000
Automatic gain control target	2×10^5
Maximum injection time	100 ms
Exclusion of isotopes	On
Dynamic exclusion time	3.0 s
Apex trigger	1 to 3 s

[a)] Maximum injection time of 75 ms was set for 240 min analyses to enhance method sensitivity.
[b)] Exclusion time in seconds was calculated as gradient time in minutes divided by two.
Only the most intense charge state of each peptide was subjected to fragmentation in all DDA experiments.

Table S3: Predicted minimum lengths of the capillary connecting the trap and separation column to avoid temperature mismatch

	**Flow rate (µl/min):**	**300**	**68**	**50**
Temperature regime trap/separation column (°C)	T_{out} **(°C):**	Required length (cm):		
22/60	**59**	15.8	3.6	2.6
22/70	**69**	16.8	3.8	2.8
22/80	**79**	17.6	4.0	2.9
35/60	**59**	14.0	3.2	2.3
35/70	**69**	15.4	3.5	2.6
35/80	**79**	16.5	3.7	2.8

The predicted length of a stainless-steel capillary to be necessarily placed in a column thermostat to preheat the 100% water mobile phase to T_{out} calculated using Equation 7 at various flow rates. A capillary passively heated in a forced-air thermostat with 0.1 mm inner and 1/16 in. outer diameter was considered. The following parameters were used: mobile phase density ρ = 1 g/mL; mean specific heat of water, C = 4.323 J/g °C and capillary resistance to heat transfer, R_{th} = 0.03 W/°C. The value of R_{th} is taken from Yan et al. [9], which is relevant for a silicone oil bath. Since we used an air bath instead of a silicone oil one as a column thermostat, the calculation includes that a roughly 7-fold longer capillary is needed to approach the same temperature using an air bath.[10] Note that the calculated length is the part of the capillary that is to be necessarily placed in a thermostat, while the entire capillary should be adequately larger to ensure the connection. T_{out} is lower than T_{therm} by 1 °C because it approaches T_{therm} asymptotically and the remaining 1 °C is the hardest to complete. It was reported that T_{out} can be up to 5 °C lower than the separation column temperature (or T_{therm}) without a noticeable drop in the separation performance.[9,11,12] Likely, this results from the frictional heating that cancels the column cooling to a small extent.[13] That is why we tolerated a little temperature mismatch, which enabled significant shortening of the connecting capillary.

Table S4: Recent publications involving peptide mapping of biopharmaceuticals

№	Title	Link	Date of publication	t_G (min)	T_{column}, (°C)	Mobile phase additive	Separation column	Detection
1	A high-resolution multi-attribute method for product characterization, process characterization, and quality control of therapeutic proteins	https://doi.org/10.1016/j.ab.2022.114575	24.01.2022	75	50	0.1% FA	Agilent Zorbax 300SB-C_{18} RR HD 1.8 µm, 2.1 × 150 mm	MS
2	Multi attribute method implementation using a High Resolution Mass Spectrometry platform: From sample preparation to batch analysis	https://doi.org/10.1371/journal.pone.0262711	27.01.2022	55	40	0.1% FA	bioZen Peptide XB-C_{18} 2.6 µm, 2.1 × 150 mm	MS
3	Automated multi-attribute method sample preparation using high-throughput buffer exchange tips	https://doi.org/10.1002/rcm.9222	30.01.2022	50	50	0.1% FA	Agilent Zorbax C_{18} 300 SB 1.8 µm, 2.1 × 150 mm	MS
4	Structural identification and absolute quantification of monoclonal antibodies in suspected counterfeits using capillary electrophoresis and liquid chromatography-tandem mass spectrometry	https://doi.org/10.1007/s00216-022-03913-y	31.01.2022	38	n.a.	0.1% FA	Acquity UPLC BEH C_{18} 1.7 µm, 2.1 × 150 mm	MS
5	Low pKa of Lys promotes glycation at one complementarity-determining region of a bispecific antibody	https://doi.org/10.1016/j.bpj.2022.02.002	15.03.2022	75	n.a.	0.05%/ 0.1% TFA	Acquity UPLC BEH C_{18}	MS
6	Development of a comprehensive approach for performance evaluation of a quantitative multi-attribute method as a quality control method	https://doi.org/10.1007/s44211-022-00090-x	17.03.2022	63	50	0.1% TFA	Agilent Zorbax 300SB-C_{18} RR HD 1.8 µm, 2.1 × 100 mm	MS
7	Identification, Efficacy, and Stability Evaluation of Succinimide Modification With a High Abundance in the Framework Region of Golimumab	https://doi.org/10.3389/fchem.2022.826923	05.04.2022	80	45	0.1% FA	Acquity UPLC BEH C_{18} 1.7 µm, 2.1 × 100 mm	MS
8	Multi-approach LC-MS methods for the characterization of species-specific attributes of monoclonal antibodies from plants	https://doi.org/10.1016/j.jpba.2022.114796	26.04.2022	120	60	0.1% TFA	Symmetry 300 C_{18} column 3.5 µm, 1.0 × 150 mm	UV + MS
9	Improvements on sample preparation and peptide separation for reduced peptide mapping based multi-attribute method analysis of therapeutic monoclonal antibodies using lysyl endopeptidase digestion	https://doi.org/10.1016/j.chroma.2022.463161	19.05.2022	45	40	0.02% TFA	Waters HSS T3 column 1.8 µm, 2.1 × 150 mm	MS
10	Optimized Methods for Analytical and Functional Comparison of Biosimilar mAb Drugs: A Case Study for Avastin, Mvasi, and Zirabev	https://doi.org/10.3390/scipharm90020036	31.05.2022	45	n.a.	0.1% FA	n.a.	MS

№	Title	Link	Date of publication	t_G (min)	T_{column}, (°C)	Mobile phase additive	Separation column	Detection
11	Characterization and Value Assignment of a Monoclonal Antibody Reference Material, NMIJ RM 6208a, AIST-MAB	https://doi.org/10.3389/fmolb.2022.842041	06.06.2022	55	45	0.1% FA	Acquity UPLC Peptide BEH C_{18} 3.5 µm, 2.1 × 150 mm	MS
12	Elucidating chemical and disulfide heterogeneities inrituximab using reduced and non-reduced peptide mapping	https://doi.org/10.1002/jssc.202200290	07.06.2022	20	55	0.1% FA	AdvanceBio Peptide Plus C_{18} column 2.7 µm, 2.1 × 150 mm	MS
13	Observation of Heavy-Chain C-Terminal Amidation in Human Endogenous IgG	https://doi.org/10.1016/j.xphs.2022.06.012	16.06.2022	77	50	0.1% FA	Acquity Peptide CSH C_{18} 1.7 µm, 2.1 × 150 mm	MS
14	In vitro/in vivo degradation analysis of trastuzumab by combining specific capture on HER2 mimotope peptide modified material and LC-QTOF-MS	https://doi.org/10.1016/j.aca.2022.340199	31.07.2022	102	60	0.1% FA	Acquity UPLC BEH C_{18} 1.7 µm, 2.1 × 150 mm	MS
15	Mass spectrometry-based multi-attribute method for mutation analysis in the early development of therapeutic proteins	https://doi.org/10.1016/j.jpba.2022.115018	23.08.2022	80	45	0.1% FA	Acquity UPLC BEH C_{18} 1.7 µm, 2.1 × 100 mm	MS
16	Maximizing hydrophobic peptide recovery in proteomics and antibody development using a mass spectrometry compatible surfactant	https://doi.org/10.1016/j.ab.2022.114924	24.09.2022	80	40	0.05%/ 0.045% TFA	Acquity UPLC BEH C_{18} 1.7 µm, 2.1 × 150 mm	MS
17	Development and optimization of a LC-MS based multi-attribute method (MAM) workflow for characterization of therapeutic Fc-fusion protein	https://doi.org/10.1016/j.ab.2022.114969	04.11.2022	47	40	0.1% FA	Shim-pack Arata peptide C_{18}	MS
18	Discovery and Control of Succinimide Formation and Accumulation at Aspartic Acid Residues in The Complementarity-Determining Region of a Therapeutic Monoclonal Antibody	https://doi.org/10.1007/s11095-022-03462-0	20.01.2023	87	60	0.1% FA	Acquity UPLC BEH C_{18} 1.7 µm, 2.1 × 150 mm	MS
19	Integrating ultra-high-performance liquid chromatography tandem mass spectrometry and imaged capillary isoelectric focusing for in-depth characterization of complex fusion proteins	https://doi.org/10.1002/rcm.9484	03.02.2023	95	n.a.	0.1% FA	Acquity UPLC BEH C_{18} 1.7 µm, 2.1 × 150 mm	MS
20	A Robust Purity Method for Biotherapeutics Using New Peak Detection in an LC–MS-Based Multi-Attribute Method	https://doi.org/10.1021/jasms.2c00355	21.02.2023	42	77	0.1% FA	Acquity Peptide CSH C_{18} 1.7 µm, 2.1 × 150 mm	MS
21	Interlaboratory Evaluation of a User-Friendly Benchtop Mass Spectrometer for Multiple-Attribute Monitoring Studies of a Monoclonal Antibody	https://doi.org/10.3390/molecules28062855	22.03.2023	60	65	0.1% TFA	Acquity UPLC BEH C_{18} 1.7 µm, 2.1 × 100 mm	MS

№	Title	Link	Date of publication	t_G (min)	T_{column}, (°C)	Mobile phase additive	Separation column	Detection
22	In-Depth Characterization of Acidic Variants Induced by Metal-Catalyzed Oxidation in a Recombinant Monoclonal Antibody	https://doi.org/10.1021/acs.analchem.2c04414	27.03.2023	40	55	0.1% FA	Acquity Peptide CSH C_{18} 1.7 µm, 2.1 × 150 mm	MS
23	A Highly Efficient Workflow for Detecting and Identifying Sequence Variants	https://doi.org/10.3390/molecules28083392	12.04.2023	85	77	0.1% FA	Acquity Peptide CSH C_{18} 1.7 µm, 2.1 × 150 mm	MS
24	A Spike-Control Approach that Evaluates High Resolution Mass Spectrometry-Based Sequence Variant Analytical Method Performance for Therapeutic Proteins	https://doi.org/10.1007/s11095-023-03527-8	01.05.2023	80	n.a.	0.1% FA	Acquity UPLC BEH C_{18} 1.7 µm, 2.1 × 150 mm	MS
25	Multi-Attribute Monitoring Method for Process Development of Engineered Antibody for Site-Specific Conjugation	https://doi.org/10.1021/jasms.3c00037	02.06.2023	90	n.a.	0.02% TFA	Acquity UPLC BEH C_{18} 1.7 µm, 2.1 × 150 mm	MS
26	Comparison of middle- and bottom-up mass spectrometry in forced degradation studies of bevacizumab and infliximab	https://doi.org/10.1016/j.jpba.2023.115596	22.06.2023	37	60	0.1% FA	AdvanceBio Peptide Map 2.7 µm, 2.1 × 250 mm	MS
27	Multi-attribute method (MAM) to assess analytical comparability of adalimumab biosimilars	https://doi.org/10.1016/j.jpba.2023.115543	22.06.2023	70	25	0.1% FA	Hypersil GOLD Vanquish C_{18} 1.9 µm, 2.1 × 150 mm	MS
28	Method validation and new peak detection for the liquid chromatography-mass spectrometry multi-attribute method	https://doi.org/10.1016/j.jpba.2023.115564	05.07.2023	70	50	0.1% FA	Agilent Zorbax C_{18} 300 SB 1.8 µm, 2.1 × 150 mm	MS
29	Expanding the Analytical Toolbox: Developing New Lys-C Peptide Mapping Methods with Minimized Assay-Induced Artifacts to Fully Characterize Antibodies	https://doi.org/10.3390/ph16091327	20.09.2023	48	77	0.1% FA	Acquity Premier Peptide CSH C_{18} 1.7 µm, 2.1 × 150 mm	MS
30	Characterization of mAb size heterogeneity originating from a cysteine to tyrosine substitution using denaturing and native LC-MS	https://doi.org/10.1016/j.jpba.2023.115743	22.09.2023	37	60	0.1% FA	AdvanceBio Peptide Map 2.7 µm, 2.1 × 250 mm	MS
31	Assessment of structural and functional similarity of biosimilar products: Bevacizumab as a case study	https://doi.org/10.1016/j.jchromb.2023.123896	25.09.2023	35	55	0.1% FA	AdvanceBio Peptide Map C_{18} 2.7 mm, 4.6 × 150 mm	UV + MS
32	Fully Unattended Online Protein Digestion and LC–MS Peptide Mapping	https://doi.org/10.1021/acs.analchem.3c01554	10.10.2023	83	50	0.1% FA	Acquity Peptide CSH C_{18} 1.7 µm, 2.1 × 150 mm	MS
33	Development, validation, and implementation of a robust and quality control-friendly focused peptide mapping method for monitoring oxidation of co-formulated monoclonal antibodies	https://doi.org/10.1007/s00216-022-04366-z	29.11.2023	28	80	0.1% FA	HALO Peptide ES-C_{18} column 2.7 µm, 2.1 × 150 mm	MS

Figure S1: Isocratic separation of small organic analytes

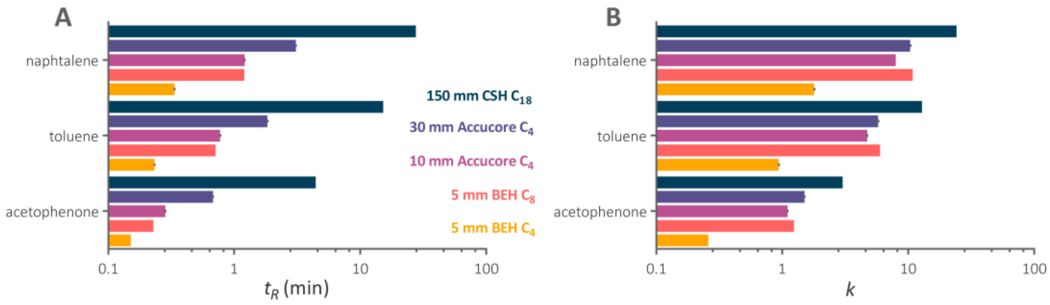

(A) Retention time (t_R) and **(B)** retention factor (k) of small organic analytes obtained from the tested 2.1 mm i.d. trap columns maintained at 22 °C and the 2.1 × 150 mm separation column maintained at 70 °C under isocratic conditions at 30% component B. Error bars represent standard deviation. Very small error bars are not visible.

Figure S2: Comparison of connecting capillaries

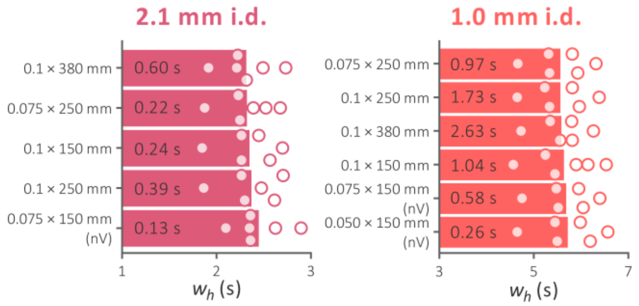

Average peak width (w_h) of iRT peptides obtained using the 2.1 and 1 mm i.d. trap-elute setups in the temperature regime of 35/80 °C assembled with various stainless steel and PEEK-shielded fused silica nanoViper (nV) capillaries. The preheater assembled on the steel 0.1 x 380 mm capillary was not actively heated. The data from the 30 min gradient are shown. Dots represent the peak width of each peptide averaged from three replicates. The first eluting peptide, LGGNEQVTR, is not included because of irreproducible retention time. Passage time through the capillaries calculated for 300 µL/min and 68 µL/min, respectively, is shown.

Figure S3: Portion of peptides effectively retained in the trap column upon injection

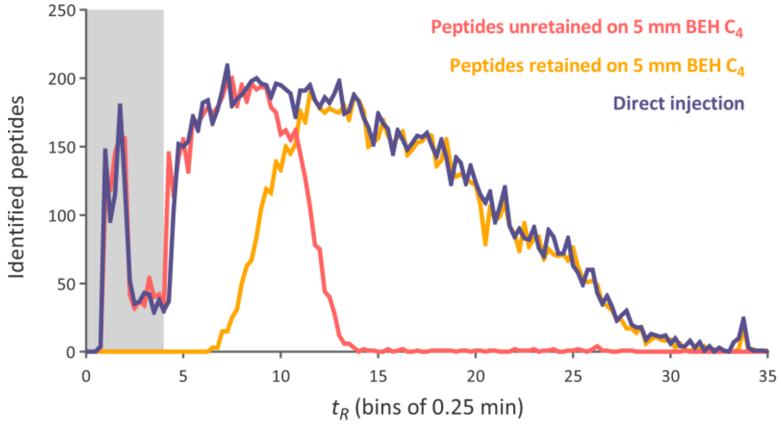

Peptides identified in 0.25-min bins during a 30-minute analysis of the *F. tularensis* digest injected using a system with a 2-position/6-port valve situated between the autosampler and separation column. The valve directed the mobile phase flow either through a 2.1 × 5 mm BEH C_4 trap column maintained at 35 °C, or through a bypass capillary. Peptides were loaded onto the trap column and isocratically eluted for 2 minutes with 0.5% component B into the separation column. The valve was then switched, and peptides retained in the separation column at 80 °C were separated using a 30-minute gradient (red). Peptides from the trap column were separated during the subsequent blank injection (yellow). For reference, peptides identified from a direct injection into the separation column with the same 2-minute isocratic hold, are also shown. The grey zone highlights peptides that were not effectively retained in either the trap or the separation column. These peptides did not benefit from the trap column but, due to their early elution, were not excessively exposed to the high separation column temperature. The percentage of peptides benefiting from the BEH C_4 trap column installation was calculated by comparing the number of peptides unretained on the trap column to those identified via direct injection. Peptides eluted in the grey zone were not considered. The experiment was conducted three times; data from the second replicate are presented.

Figure S4: Redundant peptide sequences and peptides identified only with artificial modifications

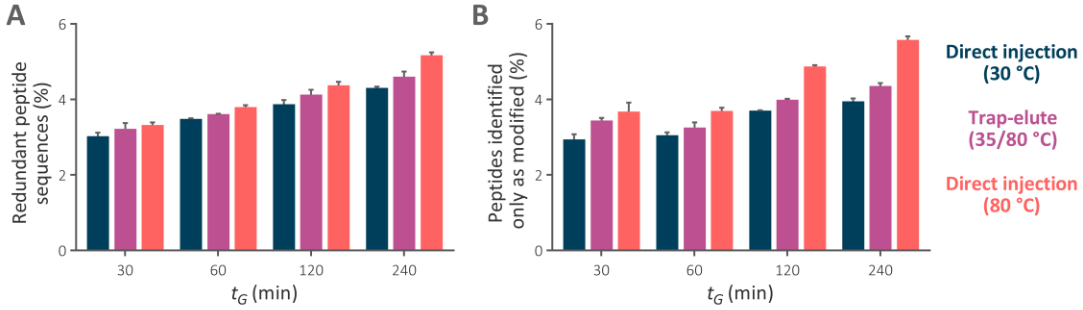

(A) The portion of peptides with a sequence that was already identified in another unmodified or modified peptide(s) and **(B)** the portion of peptides identified only in the modified form(s) in analyses of the Jurkat cells digest performed using the 1 mm i.d. trap-elute setup (35/80 °C) and direct injection in the column maintained at 30 and 80 °C.

Figure S5: Peak broadening due to the trap column installation

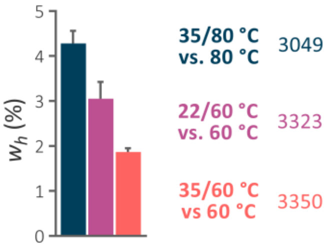

Relative increase in w_h values of peptides from the Jurkat cells digest due to the trap column installation. The peptides (300 ng) were separated using direct injection in the 1 mm i.d. separation column maintained at 60 and 80 °C and the 1 mm i.d. trap-elute setup under temperature regimes of 35/80 °C, 22/60 °C, and 35/60 °C. The apQuant node implemented in Proteome Discoverer was used to extract w_h of peptide peaks.[14] The peptides with no modification related to column temperature with <10% RSD of w_h detected in all replicates with and without the trap column were considered. Their numbers are shown right to the temperature regime.

Figure S6: Abundance of artifacts in analyses using the optimized temperature setting

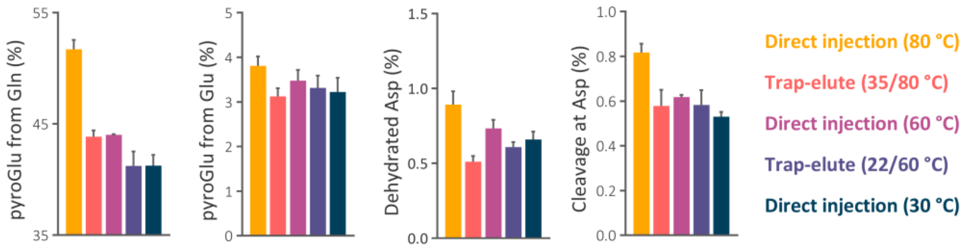

Relative abundance of the temperature-related artifacts in the 60 min analyses of the Jurkat cells digest performed using the 1 mm i.d. trap-elute setup in the temperature regimes of 35/80 °C and 22/60 °C and direct injection in the separation column maintained at 30, 60, and 80 °C.

Figure S7: Significance of in-column artificial modification for peptides of monoclonal antibody

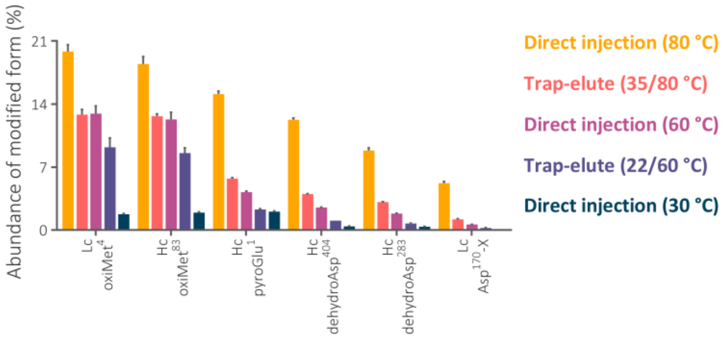

Quantity of the top 6 most abundant temperature-related peptide artifacts traced in the 110 min analyses of trastuzumab using the 2.1 mm i.d. trap-elute setup (temperature regimes of 22/60 and 35/80 °C) and direct injection in the 2.1 × 150 mm separation column maintained at 30, 60, and 80 °C. The abundance was calculated as the peak area of the modified peptide form divided by the summed peak area of both peptide forms. The most abundant pairs of parent and modified peptides containing the modified amino acid were used. Fully tryptic peptides without missed cleavages were used preferentially. The peak area was calculated from the most intense precursor charge state. Artifacts that co-eluted with unmodified peptides and exhibited no dependence on column temperature were likely generated in the ion source and not considered.

Figure S8: Peak capacity and peak width distribution in model analyses of trastuzumab

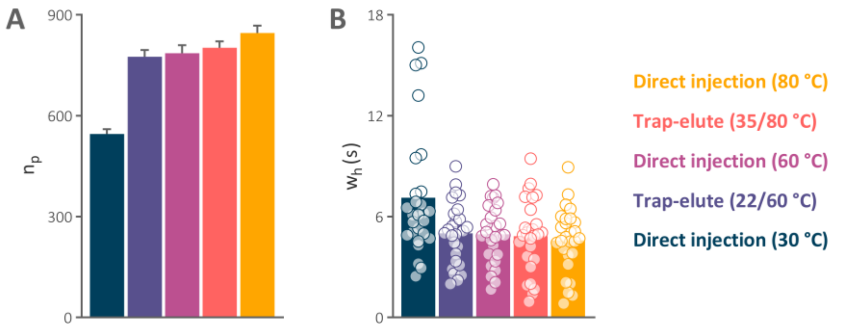

(A) Peak capacity (n_p) calculated for 110 min separation of trastuzumab tryptic peptides using the 2.1 mm i.d. trap-elute setup at temperature regimes of 22/60 °C and 35/80 °C and the direct injection in the separation column maintained at 30, 60 and 80 °C. The average peak widths of 27 unmodified fully tryptic peptides, excluding the peptides prone to in-column modification at high temperatures, were used for the calculation. **(B)** Distribution of peptide peak widths in each temperature setting.

Figure S9: Illustrative chromatograms with and without the trap column

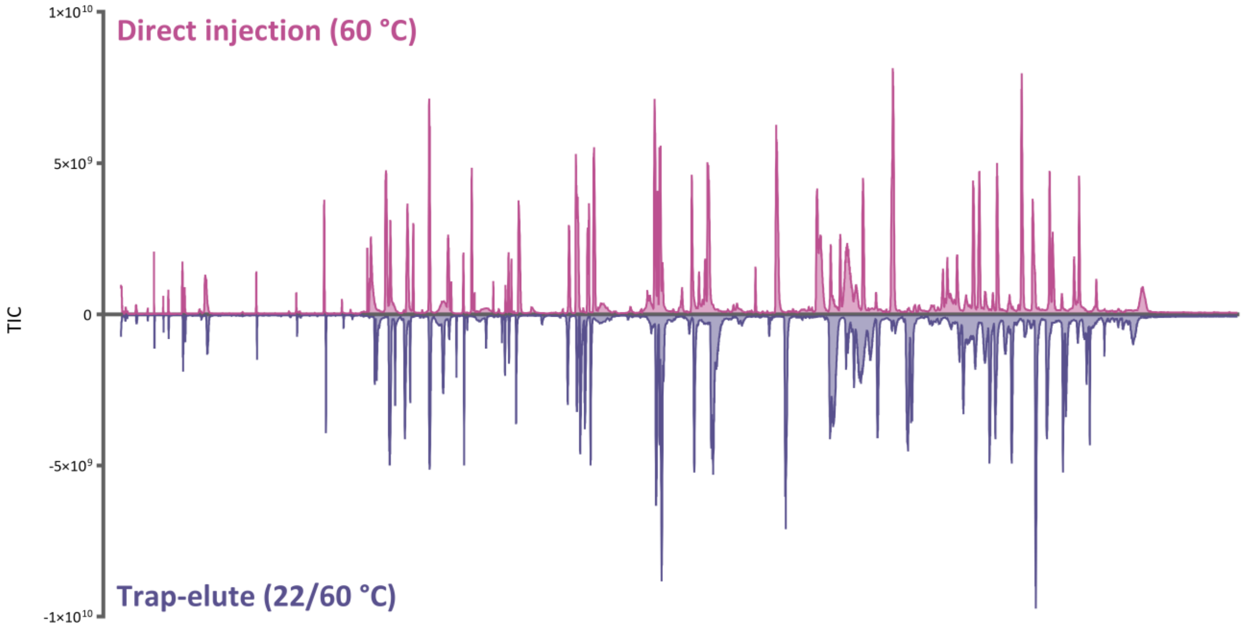

Representative chromatograms from the 110 min analyses of trastuzumab using the 2.1 mm i.d. trap-elute setup at the temperature regime of 22/60 °C and direct injection in the 2.1 × 150 mm separation column maintained at 60 °C.

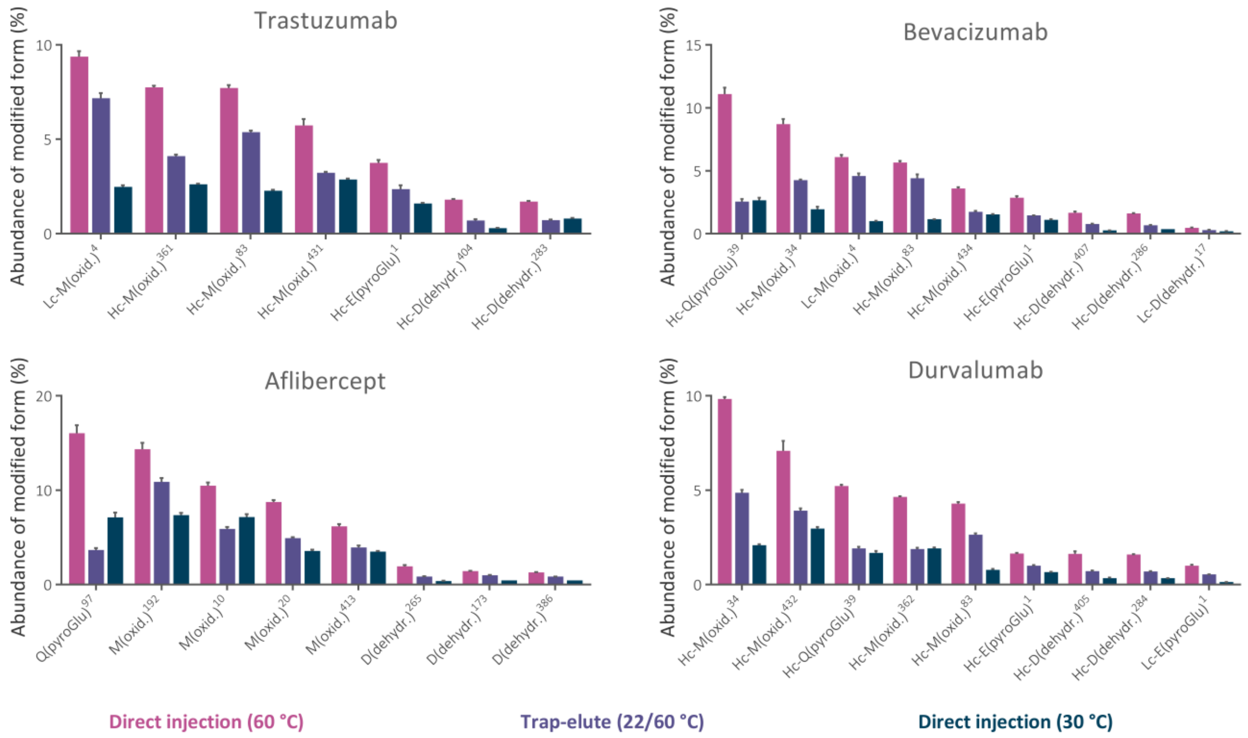

Quantity of the most abundant temperature-related peptide artifacts traced in the 90 min peptide mapping of protein biopharmaceuticals using the 2.1 mm i.d. trap-elute setup at the temperature regime of 22/60 °C and direct injection in the 2.1 × 150 mm separation column maintained at 30 and 60 °C. The abundance was calculated as the peak area of the modified peptide form divided by the summed peak area of both peptide forms. The most abundant pairs of parent and modified peptides containing the modified amino acid were used. Fully tryptic peptides without missed cleavages were used preferentially. The peak area was calculated from the most intense precursor charge state. Artifacts that co-eluted with unmodified peptides and exhibited no dependence on column temperature were likely generated in the ion source and not considered. Nonenzymatic cleavage at Asp was not considered because it can occur only artificially. Although peptides with pyroGlu at Glu/Gln located outside of the protein N-terminus fulfill the same condition, they are still illustrated because they appear as a noticeable extra peak of high peak area variability, reducing the concentration of a parent peptide.

Supporting References

(1) Escher, C.; Reiter, L.; MacLean, B.; Ossola, R.; Herzog, F.; Chilton, J.; MacCoss, M. J.; Rinner, O. *Proteomics* **2012**, *12*, 1111-1121.
(2) Stejskal, K.; Potesil, D.; Zdrahal, Z. *J Proteome Res* **2013**, *12*, 3057-3062.
(3) Smith, P. K.; Krohn, R. I.; Hermanson, G. T.; Mallia, A. K.; Gartner, F. H.; Provenzano, M. D.; Fujimoto, E. K.; Goeke, N. M.; Olson, B. J.; Klenk, D. C. *Anal Biochem* **1985**, *150*, 76-85.
(4) Zhou, J.; Zhou, T.; Cao, R.; Liu, Z.; Shen, J.; Chen, P.; Wang, X.; Liang, S. *J Proteome Res* **2006**, *5*, 2547-2553.
(5) Lenco, J.; Khalikova, M. A.; Svec, F. *J Proteome Res* **2020**, *19*, 993-999.
(6) Wisniewski, J. R.; Zettl, K.; Pilch, M.; Rysiewicz, B.; Sadok, I. *Anal Chim Acta* **2020**, *1100*, 131-137.
(7) Hains, P. G.; Robinson, P. J. *Journal of Proteome Research* **2017**, *16*, 3443-3447.
(8) Kuznetsova, K. G.; Levitsky, L. I.; Pyatnitskiy, M. A.; Ilina, I. Y.; Bubis, J. A.; Solovyeva, E. M.; Zgoda, V. G.; Gorshkov, M. V.; Moshkovskii, S. A. *J Proteomics* **2021**, *231*, 104022.
(9) Yan, B.; Zhao, J.; Brown, J. S.; Blackwell, J.; Carr, P. W. *Analytical Chemistry* **2000**, *72*, 1253-1262.
(10) Thompson, J. D.; Brown, J. S.; Carr, P. W. *Analytical Chemistry* **2001**, *73*, 3340-3347.
(11) Poppe, H.; Kraak, J. C. *Journal of Chromatography A* **1983**, *282*, 399-412.
(12) Poppe, H.; Kraak, J. C.; Huber, J. F. K.; van den Berg, J. H. M. *Chromatographia* **1981**, *14*, 515-523.
(13) Wolcott, R. G.; Dolan, J. W.; Snyder, L. R.; Bakalyar, S. R.; Arnold, M. A.; Nichols, J. A. *Journal of Chromatography A* **2000**, *869*, 211-230.
(14) Doblmann, J.; Dusberger, F.; Imre, R.; Hudecz, O.; Stanek, F.; Mechtler, K.; Durnberger, G. *J Proteome Res* **2019**, *18*, 535-541.
